# Supplementary material for: An asymmetric nautilus-like HflK/C assembly controls FtsH proteolysis of membrane proteins
Source: EMBO J. 2025 Mar 13;44(9):2501–13. doi: 10.1038/s44318-025-00408-1 (PMC12048511; doi:10.1038/s44318-025-00408-1)
Supplement: Supplementary file 1 — Appendix [file 44318_2025_408_MOESM1_ESM.pdf]

Appendix for "*An asymmetric nautilus-like HflK/C assembly controls FtsH proteolysis of membrane proteins*"

Alireza Ghanbarpour[1,2,*], Bertina Telusma[1], Barrett M. Powell[1], Jia Jia Zhang[1], Isabella Bolstad[1], Carolyn Vargas[3-5], Sandro Keller[3-5], Tania A. Baker[1], Robert T. Sauer[1,*], and Joseph H. Davis[1,6*]

[1]Department of Biology, [6]Program in Computational and Systems Biology, Massachusetts Institute of Technology, Cambridge MA, USA 02129

[2]Department of Biochemistry and Molecular Biophysics, Washington University in St. Louis, St. Louis, MO 63110

[3]Biophysics, Institute of Molecular Biosciences (IMB), NAWI Graz, University of Graz, Humboldtstr. 50/III, 8010 Graz, Austria

[4]Field of Excellence BioHealth, University of Graz, Graz, Austria

[5]BioTechMed-Graz, Graz Austria

* Correspondence – Joseph H. Davis (jhdavis@mit.edu), Robert T. Sauer (bobsauer@mit.edu), Alireza Ghanbarpour (alirezag@wustl.edu)

Table of contents

Appendix Figure S1. Domain organization of HflK, HflC, and FtsH 3

Appendix Figure S2. Purification of an active FtsH•HflK/C complex using DDM detergent 4

Appendix Figure S3. Image processing workflow for DDM-solubilized, affinity-purified FtsH•HflK/C complex 5

Appendix Figure S4. Resolution estimates for the DDM-solubilized FtsH•HflK/C structure (map a) 6

Appendix Figure S5. Density map of a GDN-solubilized FtsH•HflK/C nautilus-like supercomplex 7

Appendix Figure S6. Tomographic collection of a purified FtsH•HflK/C sample 8

Appendix Figure S7. Image processing workflow for local refinement of HflK/C 'hat-like' structure 9

Appendix Figure S8. Resolution estimates for the locally refined HflK/C hat-like structure 10

Appendix Figure S9. Resolution estimates for the FtsH•HflK/C structure bearing one FtsH hexamer per HflK/C assembly 11

Appendix Figure S10. Resolution estimates for the FtsH•HflK/C structure bearing two FtsH hexamers per HflK/C assembly 12

Appendix Figure S11. Image processing workflow for HflK/C complex affinity purified from cells overexpressing HflC-FLAG and HflK 13

Appendix Figure S12. Resolution estimates for the FtsH-free HflK/C structure 14

Appendix Figure S13. Image processing workflow for FtsH•HflK/C complex affinity-purified via detergent-free extraction 15

Appendix Figure S14. Resolution estimates for the carboxy-DIBMA-extracted FtsH•HflK/C structure 16

Appendix Figure S15. Assays of proteoliposome integrity 17

Appendix Table S1. Data-independent acquisition (DIA) windows 18

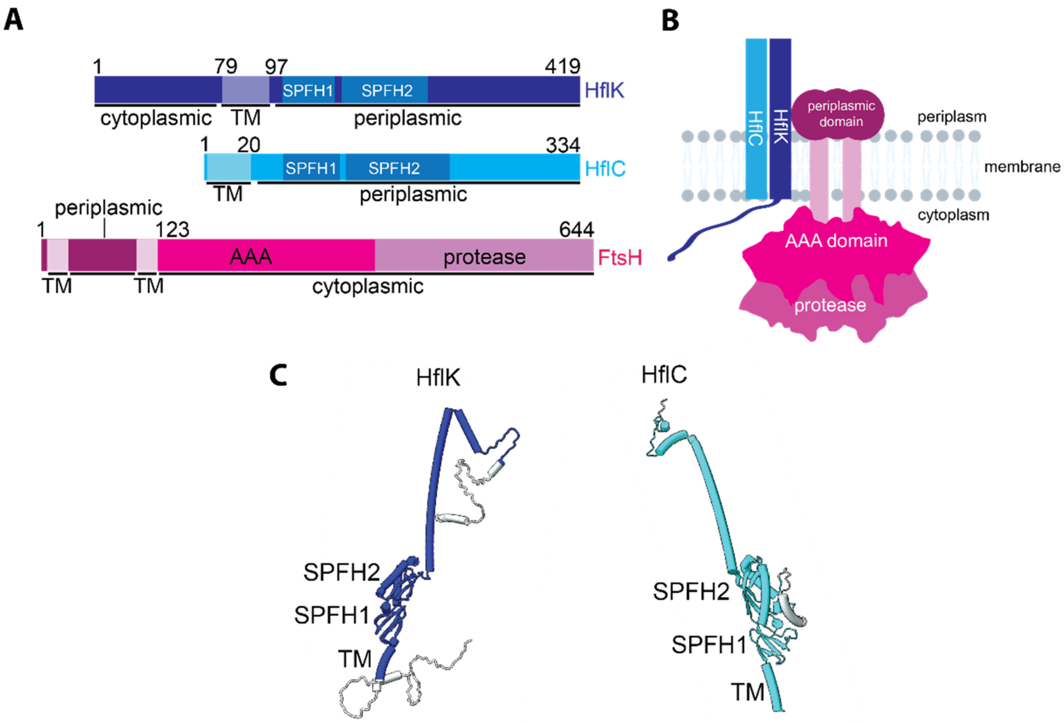

Appendix Figure S1. Domain organization of HflK, HflC, and FtsH.

(A) Domain boundaries of HflK (purple), HflC (blue), and FtsH (pink).

(B) Cartoon depicting FtsH and HflK/C domains relative to the inner membrane of *E. coli*. Note that only two of 24 HflK/C subunits are shown for simplicity.

(C) The AlphaFold models of HflK and HflC show the regions of HflK (residues 79-355) and HflC (residues 1-160 and 191-329) that were modeled in our structure, depicted in blue and cyan, respectively. The regions not modeled in our structure are shown in gray.

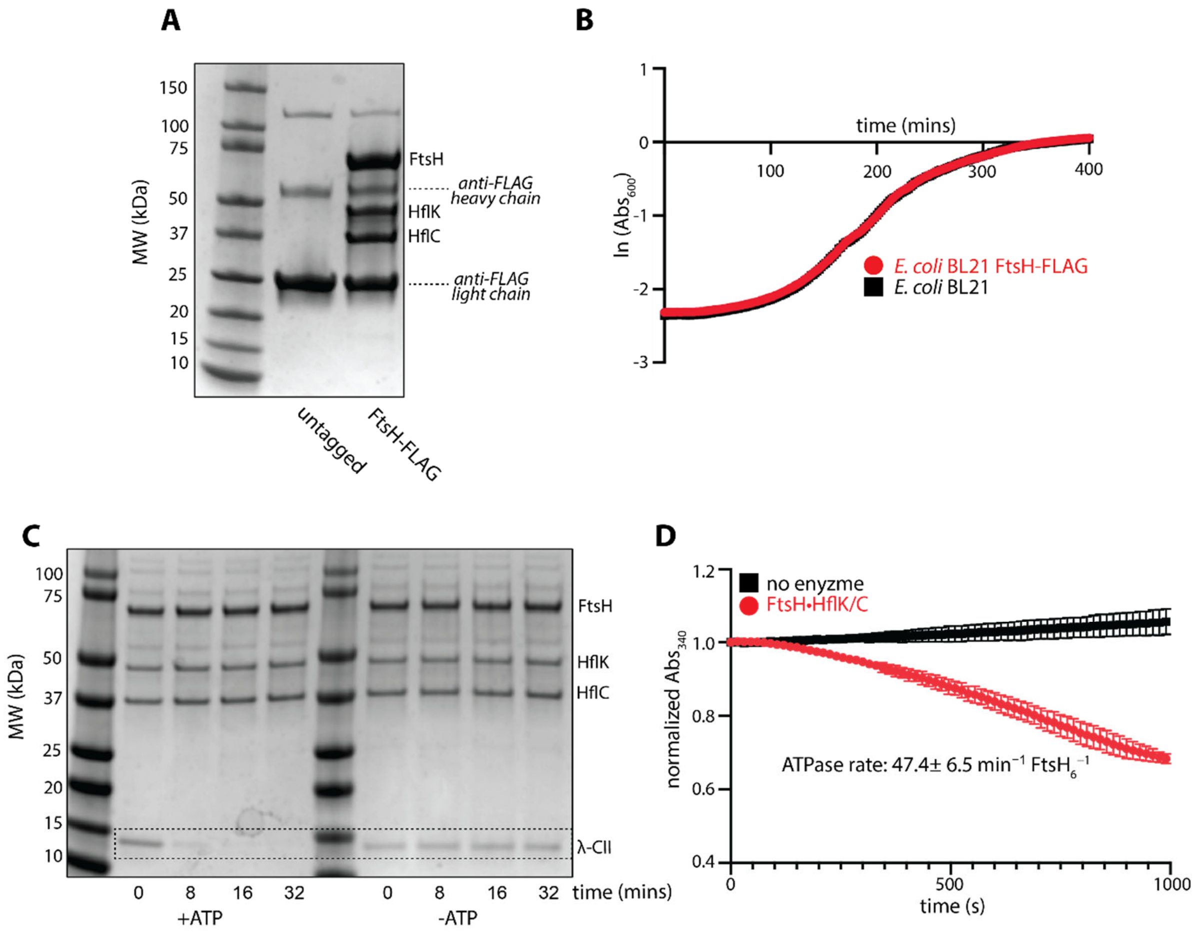

Appendix Figure S2. Purification of an active FtsH•HflK/C complex using DDM detergent. (A) SDS-PAGE of affinity-purified FtsH•HflK/C (right lane), with molecular-weight standards (left lane), and the control M2-FLAG antibody (middle lane).

(B) Rates of *E. coli* cell growth at 37°C as measured by absorbance at 600 nm. Parental BL21(DE3) strain (black) compared to that bearing a FLAG-tag fused to the C-terminus of FtsH at the endogenous genomic locus (red).

(C) SDS-PAGE assay of the degradation of λ-CII protein (5 µM) by affinity-purified FtsH•HflK/C (~0.3 µM $FtsH_6$) measured in the presence (left) or absence (right) of 5 mM ATP.

(D) ATPase assay of affinity purified FtsH•HflK/C (~0.5 µM $FtsH_6$) measured using a NADH-coupled enzymatic assay (Norbe 1988), which follows the change in absorbance at 340 nm. The linear portion of the curve was fit, producing an apparent ATPase rate of ~47 min^{-1} $FtsH^{-1}$. The assay was performed in triplicate, with mean (mark) and standard error of the mean (bars) depicted.

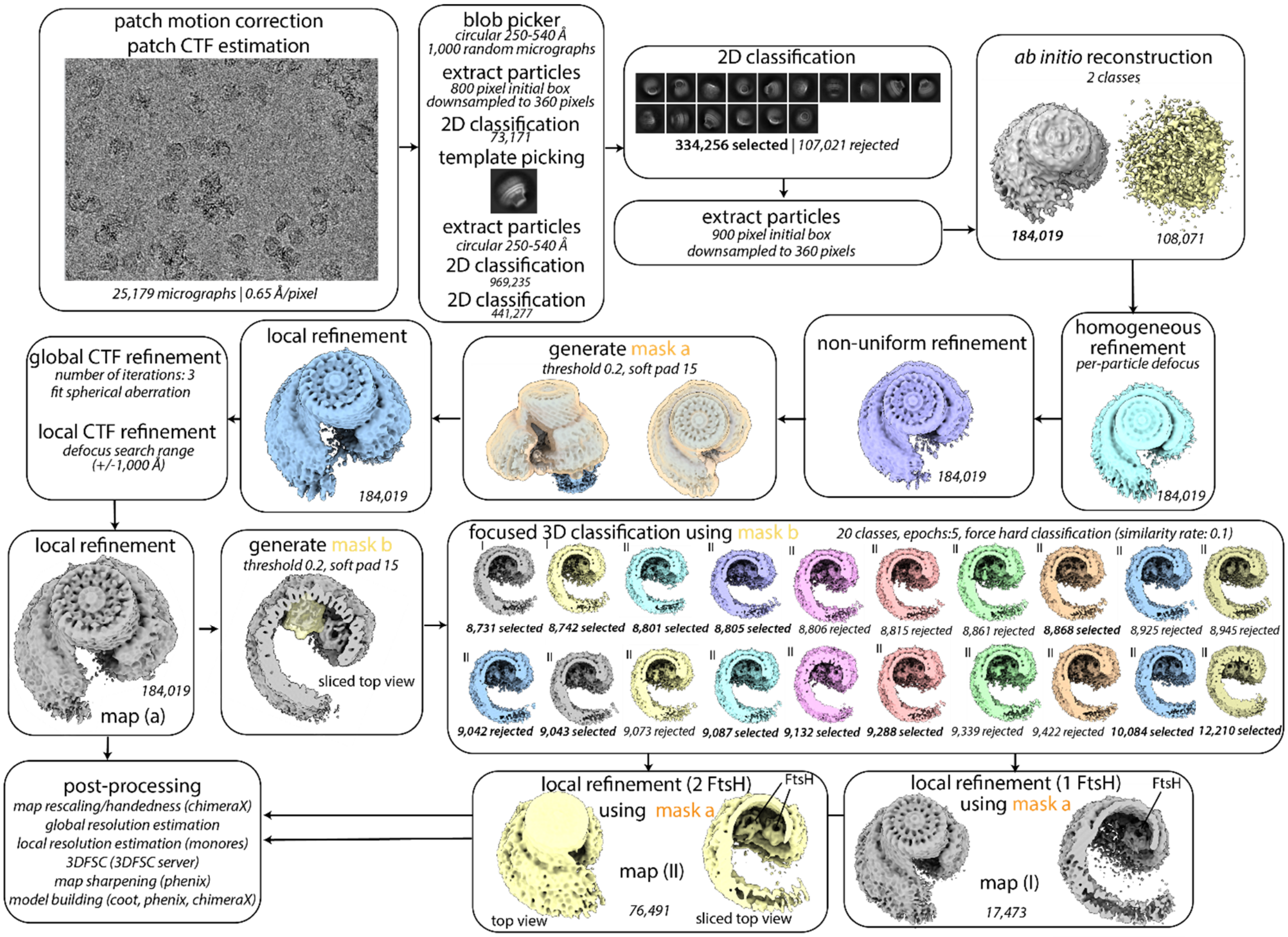

Appendix Figure S3. Image processing workflow for DDM-solubilized, affinity-purified FtsH•HflK/C complex.

Processing workflow as executed in cryoSPARC, with job names, job details, and non-default parameters (italicized) noted in each box. Map a (presented in Figure 1 and analyzed in Appendix Figure S4) was used for local refinement of the HflK/C hat-like structure as depicted in Appendix Figures S7-S8. Focused 3D classification using mask b was used to identify particles with either one (map I) or two (map II) FtsH hexamers within the HflK/C assembly. These maps are analyzed in Appendix Figures S9 and S10, respectively. Note that some particles with two FtsH hexamers were rejected during focused 3D classification due to the non-uniform conformation of the second FtsH hexamer.

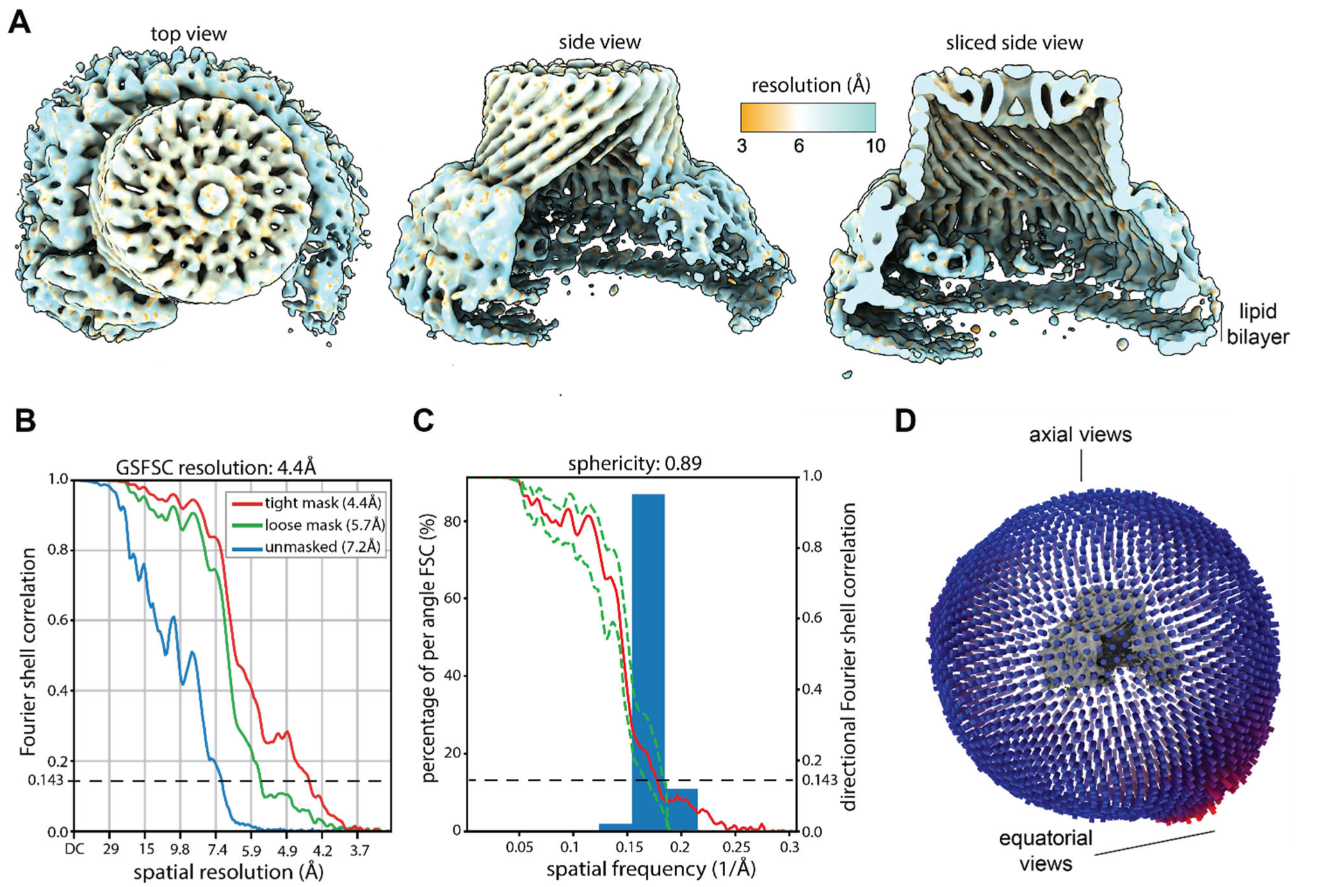

Appendix Figure S4. Resolution estimates for the DDM-solubilized FtsH•HflK/C structure (map a).

(A) Density map colored according to local resolution as estimated by a cryoSPARC implementation of MonoRes (Vilas *et al.,* 2018).

(B) Global resolution estimated by the gold-standard Fourier shell correlation method used in cryoSPARC.

(C) Directional FSC as estimated by the 3DFSC server using the 'loose' mask from (B).

(D) Projection-angle distribution.

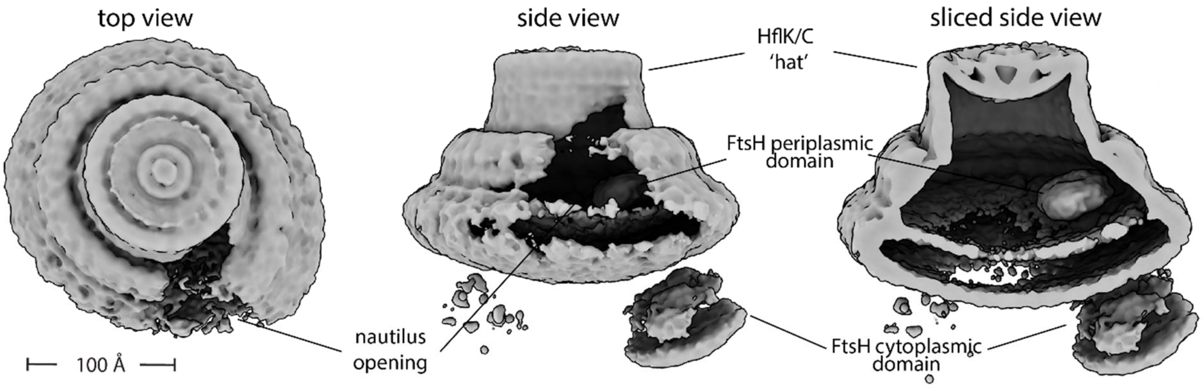

Appendix Figure S5. Density map of a GDN-solubilized FtsH•HflK/C nautilus-like supercomplex. Side, top, and sliced side views of the map are depicted, with key structural features and scale bar annotated.

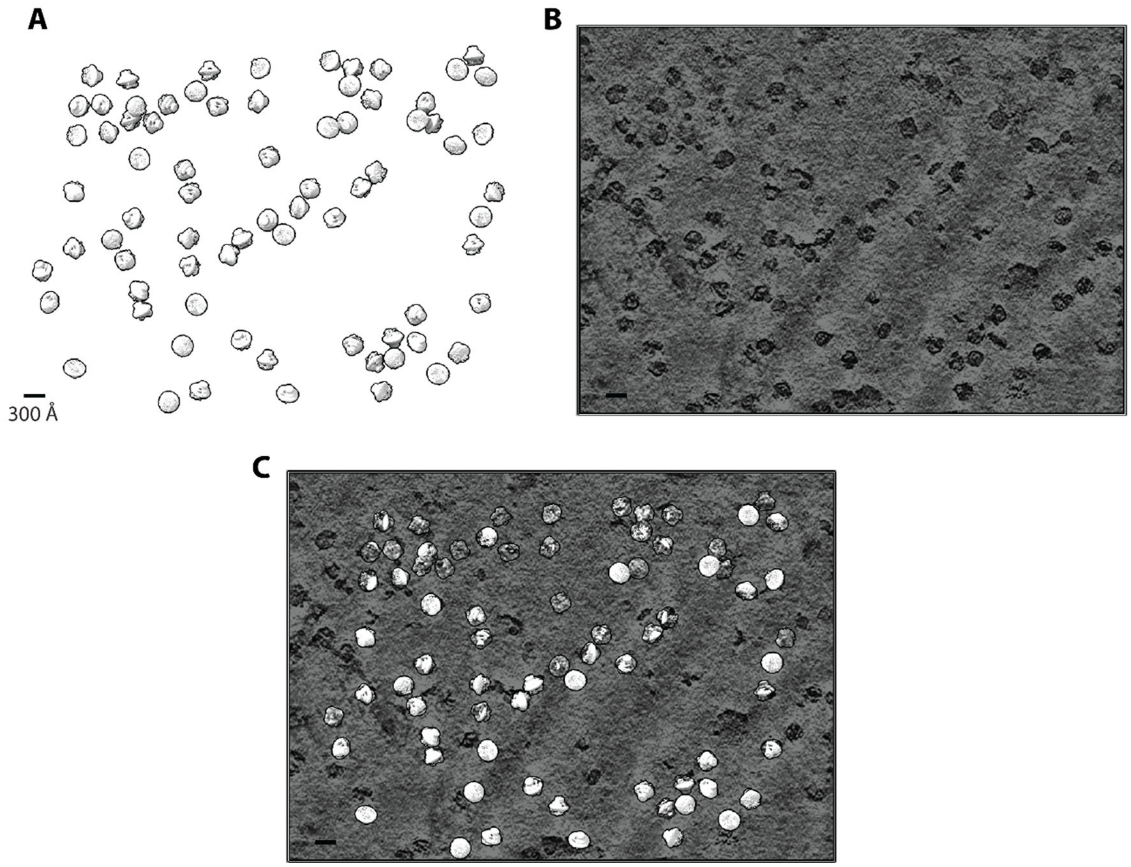

Appendix Figure S6. Tomographic collection of a purified FtsH•HflK/C sample.

(A) Tomographic reconstruction (see Methods) of a GDN-solubilized FtsH•HflK/C sample. The reconstruction is placed and oriented at the locations of observed particles in a representative tomogram. Note that particles are randomly oriented on the grid. Scale bar (300Å) indicated.

(B) Slice extracted from a the representative denoised tomogram from panel (a). Scale bar is as in panel (a).

(C) Overlay of partially-transparent tomogram slice from panel (b) with sub-tomogram averaging reconstruction from panel (a). Scale bar is as in panel (a).

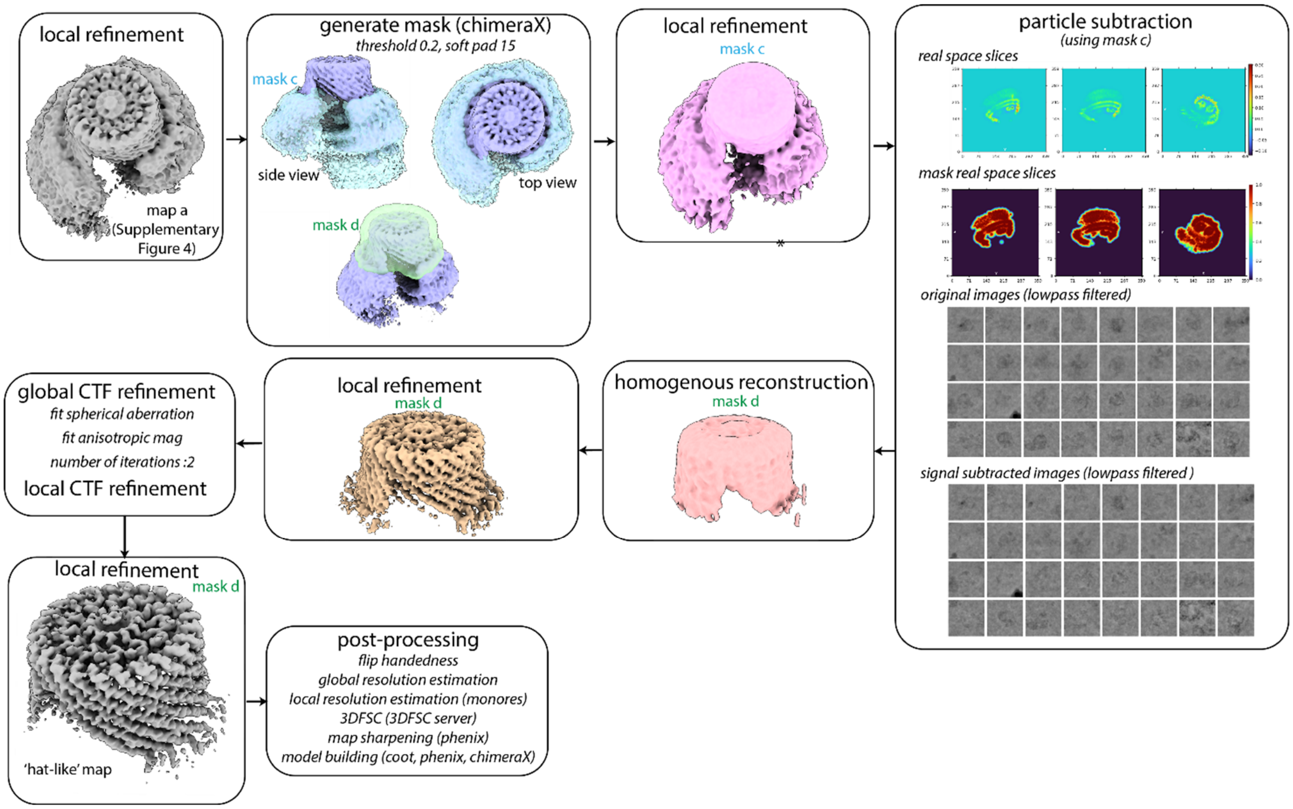

Appendix Figure S7. Image processing workflow for local refinement of HflK/C 'hat-like' structure. CryoSPARC processing workflow for the 'hat-like' portion of the HflK/C complex. Job names, job details, and non-default parameters (italicized) are noted in each box.

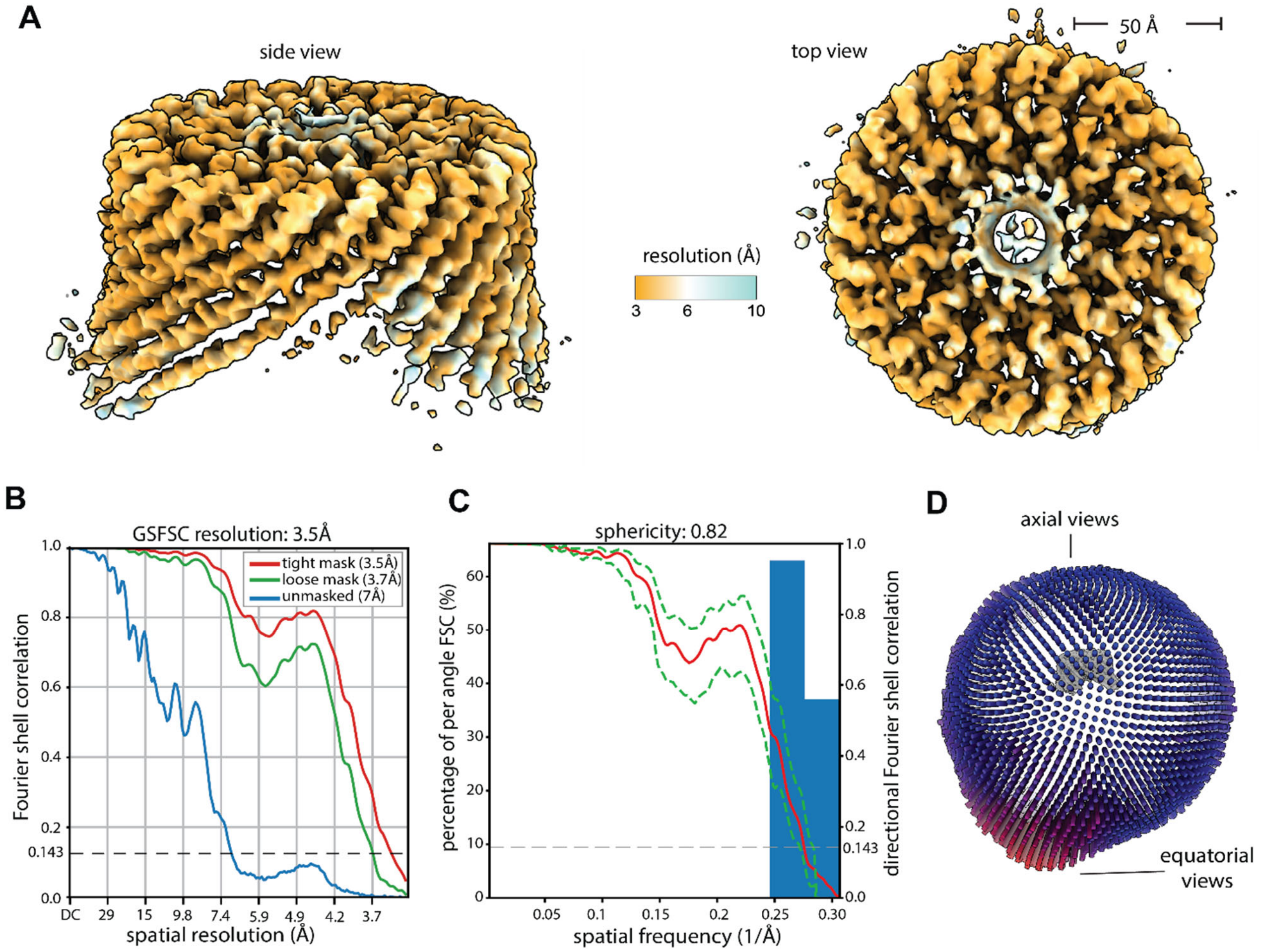

Appendix Figure S8. Resolution estimates for the locally refined HflK/C hat-like structure.

(A) Density map colored according to local resolution as estimated by a cryoSPARC implementation of MonoRes (Vilas et al, 2018).

(B) Global resolution estimated by the gold-standard Fourier shell correlation method used in cryoSPARC.

(C) Directional FSC as estimated by the 3DFSC server using the ‘loose’ mask from (B).

(D) Projection-angle distribution.

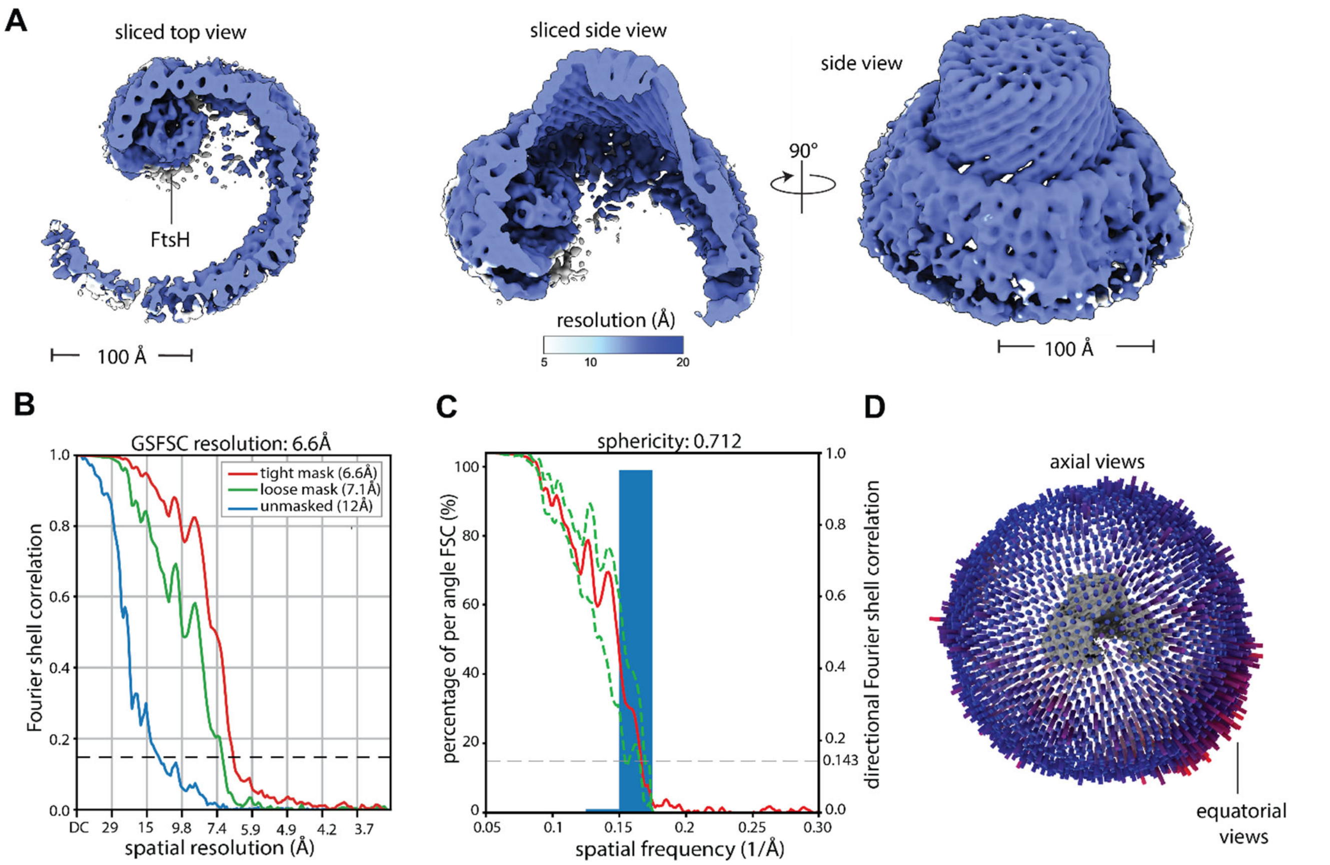

Appendix Figure S9. Resolution estimates for the FtsH•HflK/C structure bearing one FtsH hexamer per HflK/C assembly.

(A) Density map colored according to local resolution as estimated by a cryoSPARC implementation of MonoRes (Vilas et al, 2018).

(B) Global resolution estimated by the gold-standard Fourier shell correlation method used in cryoSPARC.

(C) Directional FSC as estimated by the 3DFSC server using the 'loose' mask from (B).

(D) Projection-angle distribution.

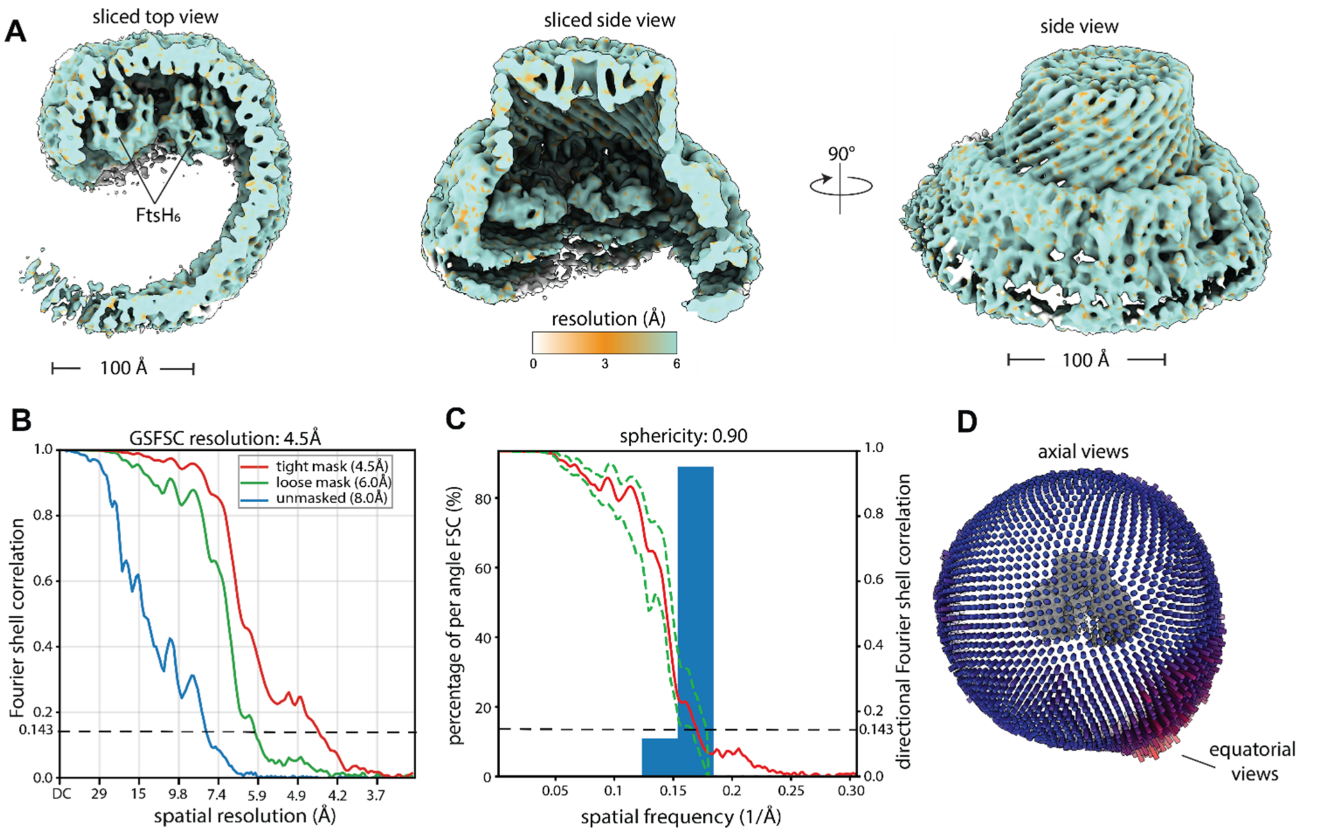

Appendix Figure S10. Resolution estimates for the FtsH•HflK/C structure bearing two FtsH hexamers per HflK/C assembly.

(A) Density map colored according to local resolution as estimated by a cryoSPARC implementation of MonoRes (Vilas et al, 2018).

(B) Global resolution estimated by the gold-standard Fourier shell correlation method used in cryoSPARC.

(C) Directional FSC as estimated by the 3DFSC server using the 'loose' mask from (B).

(D) Projection-angle distribution.

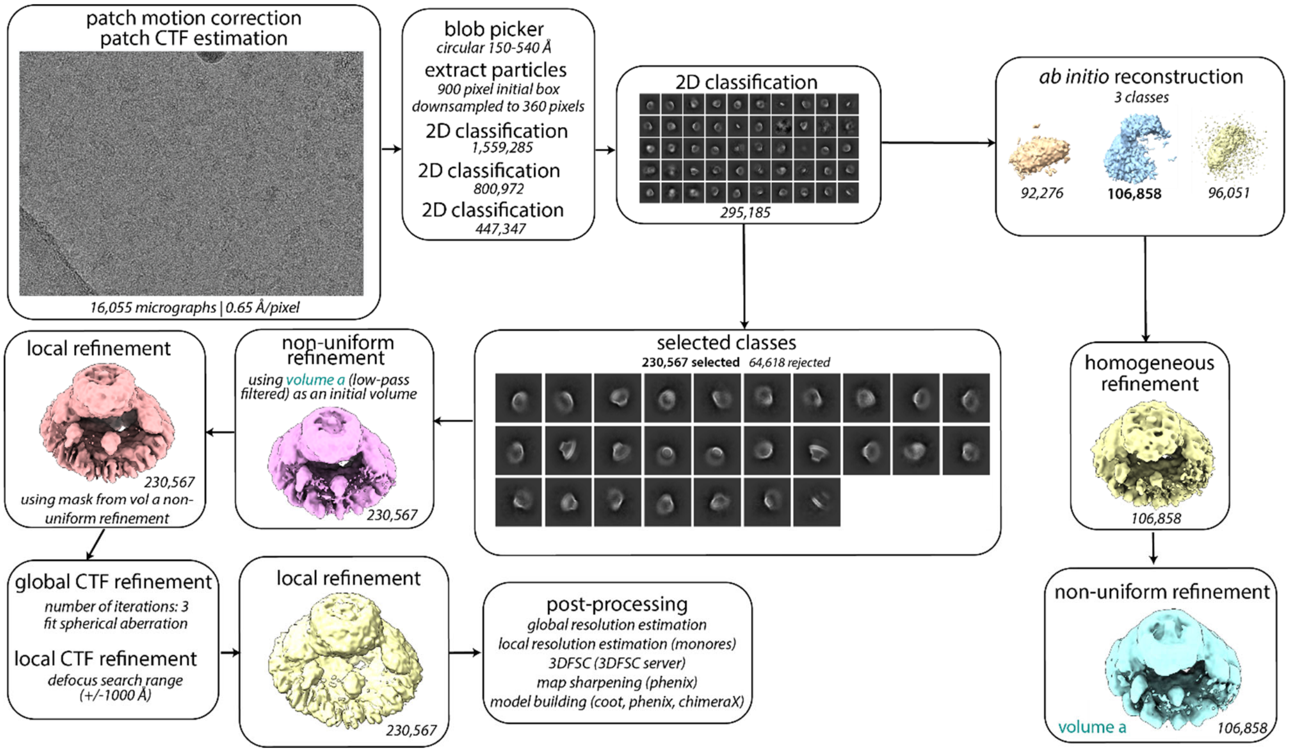

Appendix Figure S11. Image processing workflow for HflK/C complex affinity purified from cells overexpressing HflC-FLAG and HflK. CryoSPARC processing workflow for the FtsH-free HflK/C complex. Job names, job details, and non-default parameters (italicized) are noted in each box.

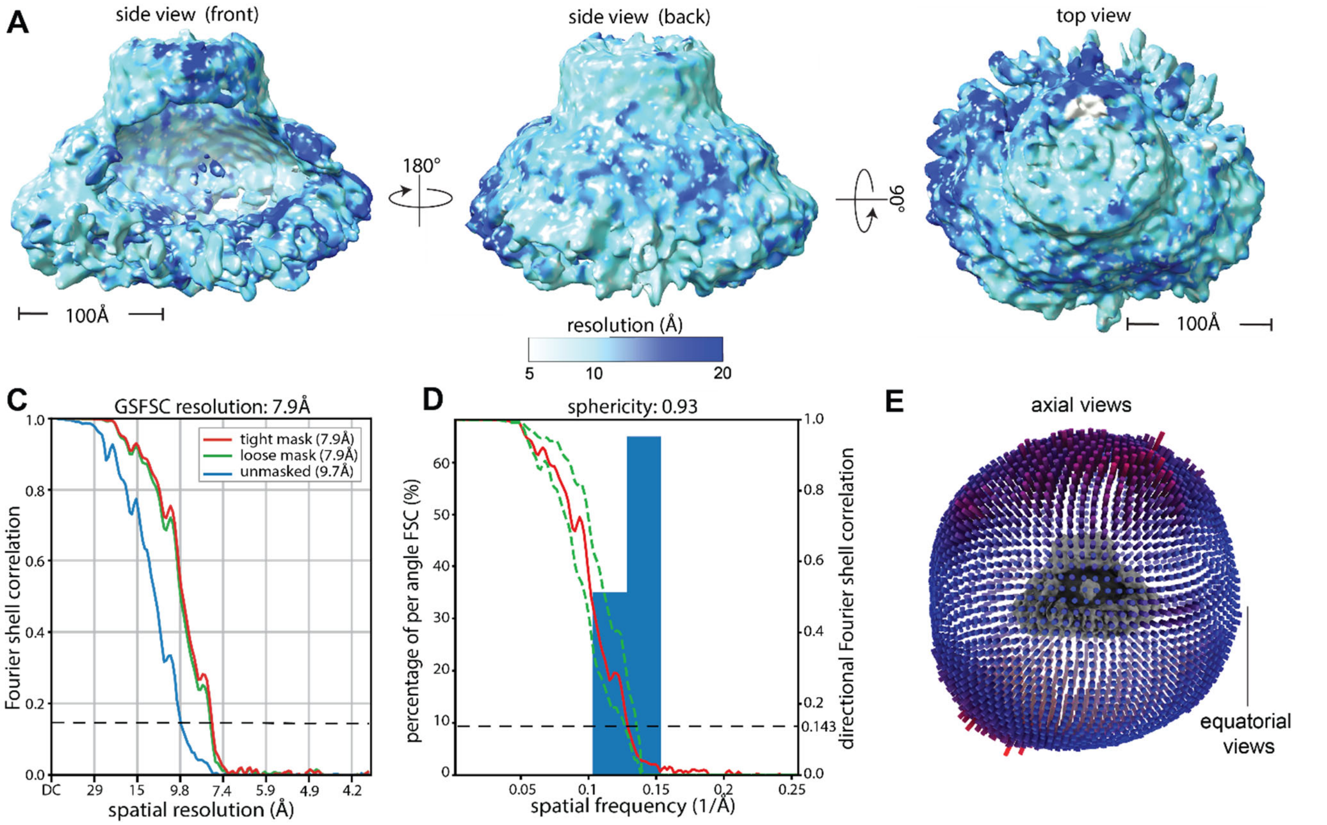

Appendix Figure S12. Resolution estimates for the FtsH-free HflK/C structure.

(A) Density map colored according to local resolution as estimated by a cryoSPARC implementation of MonoRes (Vilas et al, 2018).

(B) Global resolution estimated by the gold-standard Fourier shell correlation method used in cryoSPARC.

(C) Directional FSC as estimated by the 3DFSC server using the 'loose' mask from (B).

(D) Projection-angle distribution.

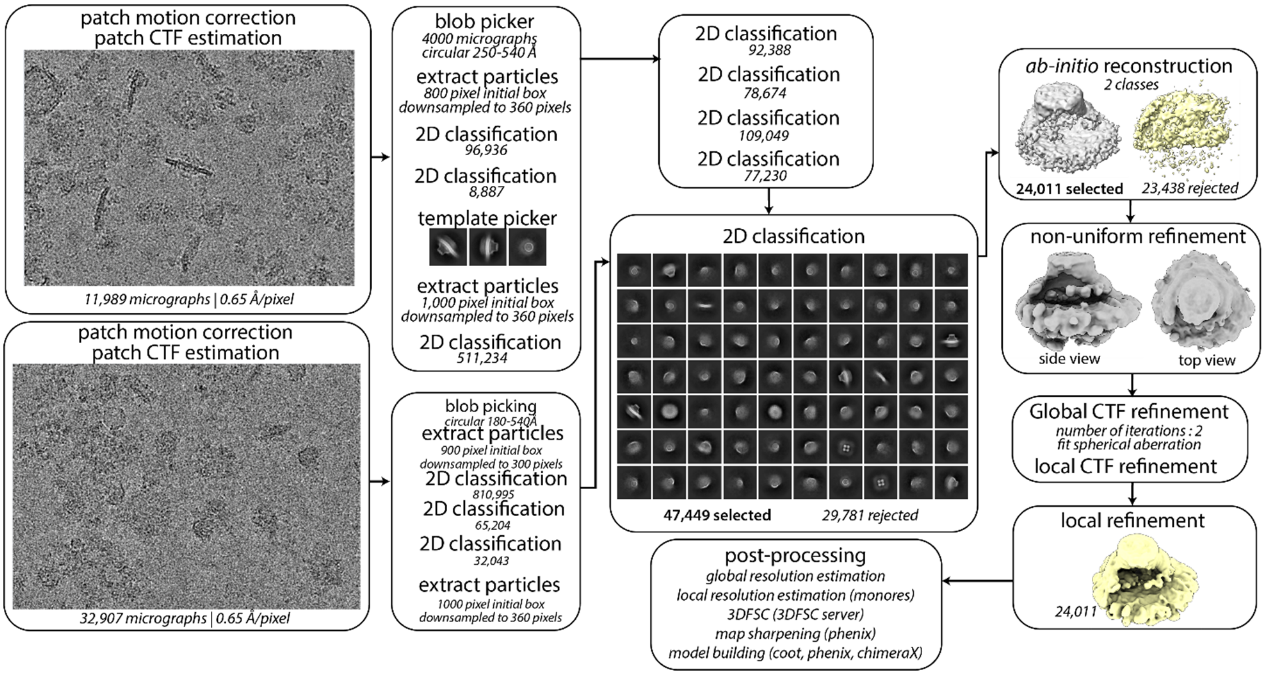

Appendix Figure S13. Image processing workflow for FtsH•HflK/C complex affinity-purified via detergent-free extraction. CryoSPARC processing workflow for the Carboxy-DIBMA-extracted FtsH•HflK/C complex. Job names, job details, and non-default parameters (italicized) are noted in each box.

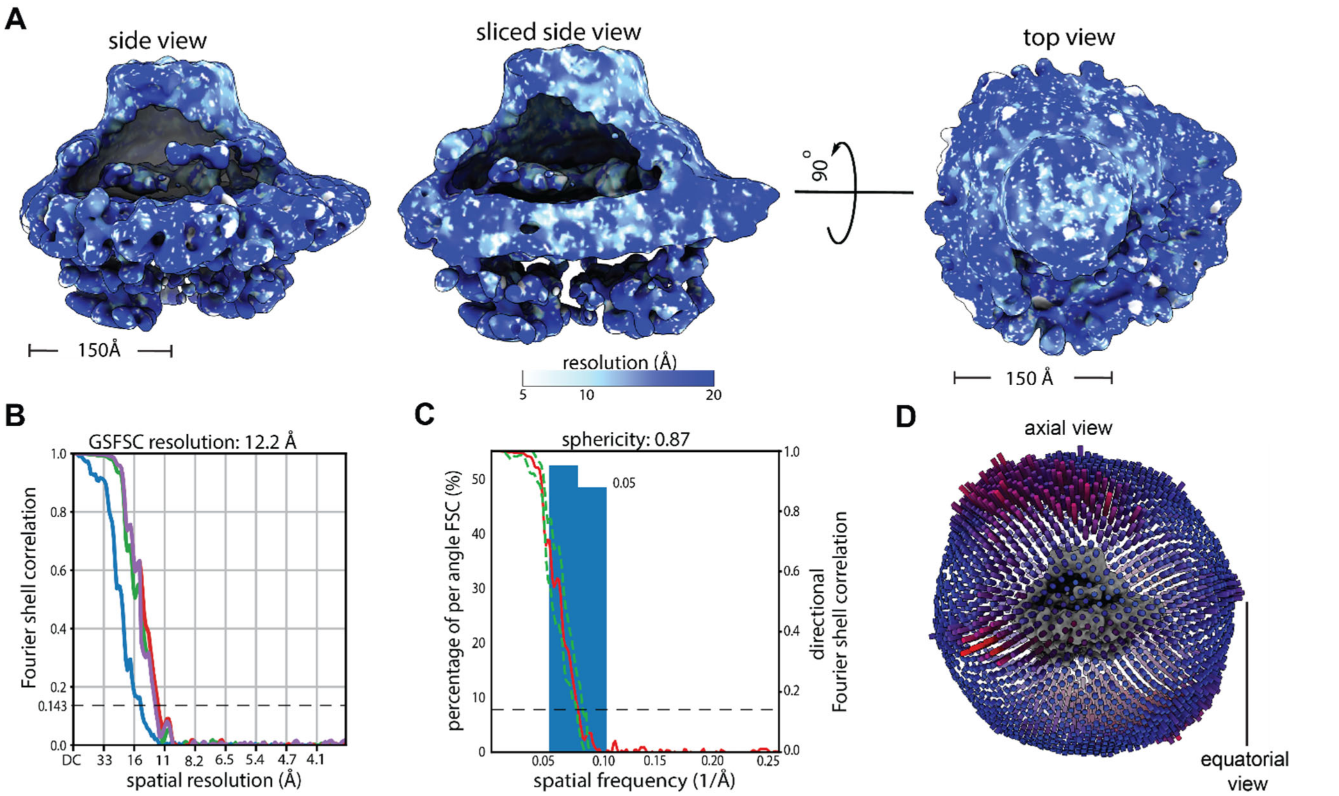

Appendix Figure S14. Resolution estimates for the Carboxy-DIBMA-extracted FtsH•HflK/C structure.

(A) Density map colored according to local resolution as estimated by a cryoSPARC implementation of MonoRes (Vilas *et al.,* 2018).

(B) Global resolution estimated by the gold-standard Fourier shell correlation method used in cryoSPARC.

(C) Directional FSC as estimated by the 3DFSC server using the 'loose' mask from (B).

(D) Projection-angle distribution.

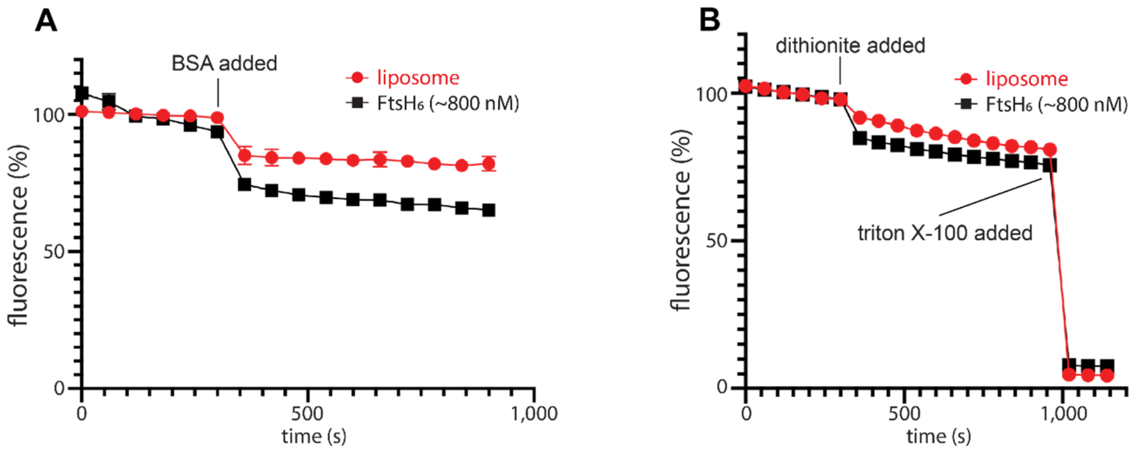

Appendix Figure S15. Assays of proteoliposome integrity.

(A) BSA back-extraction assay (Ghanbarpour *et al.,* 2021), in which BSA is added to liposomes (red) or proteoliposomes bearing $FtsH_6$ (black) to extract and quench accessible lipids.

(B) Unlabeled liposomes were formed in the presence of NBD-glucose, effectively trapping the dithionite-sensitive NBD-glucose (see Methods). Addition of dithionite had a minimal impact on measured fluorescence of either protein-free liposomes (red) or those bearing $FtsH_6$ (black), consistent with largely intact proteoliposomes.

window #	start m/z	end m/z
1	399.4315	433.4315
2	433.4469	459.4469
3	459.4587	481.4587
4	481.4688	501.4688
5	501.4778	521.4778
6	521.4869	539.4869
7	539.4951	557.4951
8	557.5033	573.5033
9	573.5106	589.5106
10	589.5178	605.5178
11	605.5251	623.5251
12	623.5333	641.5333
13	641.5415	659.5415
14	659.5497	679.5497
15	679.5588	699.5588
16	699.5679	721.5679
17	721.5779	745.5779
18	745.5888	769.5888
19	769.5997	797.5997
20	797.6124	827.6124
21	827.6261	861.6261
22	861.6415	901.6415
23	901.6597	957.6597
24	957.6852	1,041.69
25	1,041.72	1,249.72

Appendix Table S1. Data-independent acquisition (DIA) windows.
Start and end m/z values for DIA acquisition windows used in this study.
